# Social, demographic, health care and co-morbidity predictors of tuberculosis mortality in Amazonas, Brazil: a multiple cause of death approach

**DOI:** 10.1371/journal.pone.0218359

**Published:** 2020-01-29

**Authors:** Vanderson de Souza Sampaio, Maria Gabriela de Almeida Rodrigues, Leila Cristina Ferreira da Silva, Daniel Barros de Castro, Patrícia Carvalho da Silva Balieiro, Ana Alzira Cabrinha, Antonio José Leal Costa

**Affiliations:** 1 Sala de Análise de Situação de Saúde, Fundação de Vigilância em Saúde do Amazonas, Manaus, Amazonas, Brazil; 2 Programa de Pós-Graduação em Medicina Tropical, Universidade do Estado do Amazonas, Manaus, Amazonas, Brazil; 3 Fundação de Medicina Tropical Dr. Heitor Vieira Dourado, Manaus, Amazonas, Brazil; 4 Núcleo de Ensino e Pesquisa, Fundação de Vigilância em Saúde do Amazonas, Manaus, Amazonas, Brazil; 5 Núcleo de Sistemas de Informações, Fundação de Vigilância em Saúde do Amazonas, Manaus, Amazonas, Brazil; 6 Área de Epidemiologia e Bioestatística, Instituto de Estudos em Saúde Coletiva, Universidade Federal do Rio de Janeiro, Rio de Janeiro, Rio de Janeiro, Brazil; Universidade Federal de Pelotas, BRAZIL

## Abstract

**Objectives:**

Estimate TB mortality rates, catalogue multiple causes on death certificates in which TB was reported and identify predictors of TB from reporting on death certificates in the State of Amazonas, Brazil, based on a multiple cause of death approach.

**Methods:**

The death records of residents in the Amazonas state between 2006–2014 were analyzed and separated into three categories: TB not reported on the death certificate (TBNoR), TB reported as the underlying cause of death (TBUC) and TB reported as an associated cause of death (TBAC). Age standardized annual mortality rates for TBUC, TBAC and with TB reported (TBUC plus TBAC) were estimated for the State of Amazonas using the direct standardization method and World Health Organization 2000–2025 standard population. Mortality odds ratios (OR) for reporting of TBUC and TBAC were estimated using multinomial logistic regression.

**Results:**

Age standardized annual TBUC and TBAC mortality rates ranged between 5.9–7.8/10^5^ and 2.7–4.0/10^5^, respectively. TBUC was associated with being a resident in the State capital (OR = 0.66), of female gender (OR = 0.87), having an education level of 8 to 11, or 12 or more school years (OR = 0.67 and 0.50 respectively), non-white race/skin color (OR = 1.38) and place of death reported as in the State capital (OR = 1.69). TBAC was related to the triennium in which death occurred (OR = 1.21 and 1.22 for the years 2009–2011 and 2012–2014 respectively), age (OR = 36.1 and 16.5 for ages 15–39 and 40–64 years respectively) and when death occurred in the State capital (OR = 5.8).

**Conclusions:**

TBUC was predominantly associated with predictors of unfavorable socioeconomic conditions and health care access constraints, whereas TBAC was mainly related to ages which were typical of high HIV disease incidence.

## Introduction

Globally, tuberculosis ranks as the ninth leading cause of death and the leading cause of death by a single infectious agent [1]. It is estimated that 10.0 million people (range, 9.0–11.1 million) developed TB in 2017 and 1.3 million of these patients died [1]. In Brazil, one of the countries considered to have a high tuberculosis burden according to the World Health Organization (WHO), 72,788 new TB cases were recorded in 2018, and in 2017 there were 4,534 deaths from the disease. Nevertheless, tuberculosis remains a public health threat on a nationwide level with markedly heterogeneous patterns of morbidity and mortality [2].

The State of Amazonas has historically been among the highest in the ranking levels for tuberculosis incidence and mortality in Brazil. For all forms of tuberculosis, the state’s incidence rate (72.9/100,000) ranked first in 2018 and, in 2017, its mortality rate (3.9/100,000) was third among Brazilian States, exceeding the corresponding national incidence (34.8/1000,000) and mortality (2.2/100,000) estimates by 2.1 and 1.8 times [2].

So far, mortality statistics based on the criteria for underlying cause of death have underpinned public health epidemiology analysis. A more comprehensive understanding of population mortality patterns requires knowledge of the myriad of causes related to the occurrence of death, as provided by multiple cause of death analysis [3]. In multiple cause of death analysis, all causes registered on death certificates are considered, allowing enumeration of the underlying cause and the associated causes, including consequential causes—those following the underlying cause and thus directly related to death–and contributing causes–those not directly related, but that in some way contributed to death [4].

Multiple cause of death analysis of tuberculosis is able to provide information on which complications follow tuberculosis when assigned as the underlying cause of death, as well as the underlying causes when tuberculosis is reported as an associated cause. It also allows a more comprehensive estimation of mortality involving tuberculosis [4].

Despite TB’s high morbidity and mortality levels, to our knowledge no studies in Brazil have focused on the factors related to tuberculosis mortality using multiple cause of death analysis.

There are studies available that have analyzed factors associated to death from tuberculosis as the underlying cause and as a result found predictors of death conditions related to the deterioration of health services, increased resistance to TB, use of antiretroviral therapy, cotrimoxazole preventive therapy (CPT), and type of TB diagnosis [5,6]. Thus, existing knowledge points out that risk factors are related to the occurrence of TB, and the sociodemographic and structural characteristics of services in the region. Therefore, it is important to know if it is possible to reduce the risk of death through the understanding of mortality predictors and by improving patient care.

Based on a multiple cause of death approach, the aim of the present study was to estimate TB mortality rates, describe multiple causes assigned on death certificates in which TB was reported and identify predictors from TB reporting on death certificates in the State of Amazonas, Brazil.

## Material and methods

Located in the northern region of Brazil, at 1,559,161 km^2^, Amazonas is Brazil’s largest State, most of it covered by the Amazon forest and its hydrographic basin. In 2010, it had a population of 3,483,985 inhabitants in 62 municipalities. 79% of this population was living in urban settings and 21% in rural areas. The average demographic density in 2010 was 2.23 inhabitants/km^2^. Only eight municipalities had more than 50,000 inhabitants. Manaus, the State capital had 52% (1,802,014) of the State’s population and better access to public services when compared to the countryside. In 29 municipalities, the population was below 20,000. Almost 5% of the population is indigenous, mainly of Tikuna ethnicity [7].

A cross-sectional exploratory study based on a series of official mortality registers was carried out. Data were obtained from the Mortality Information System (Sistema de Informações sobre Mortalidade–SIM, available at http://datasus.saude.gov.br/) coordinated by the State of Amazonas Foundation for Public Health Surveillance (Fundação de Vigilância em Saúde do Estado do Amazonas–FVS AM). Data included into the SIM derives from a nationwide standard death certificate filled in by physicians, containing demographic, socio-economic, health assistance and cause of death data. Automated selection of underlying cause of death has been in use in Brazil since 1996 in state and municipal health departments, and follows the 10^th^ revision of the International Classification of Diseases (ICD10) coding rules for selection and modification [8]. Both underlying and associated causes registered in parts I and II of the international form for medical certificate of cause of death have been recorded in the SIM since 1996. Associated causes include both consequential (terminal and intervening) and contributing causes of death [9]. All causes registered on death certificates, including symptoms, signs, and modes of death were included.

Non-fetal death records of residents in the state of Amazonas where death occurred within the State from January 1^st^, 2006 until December 31^st^, 2014 were included in the study after eliminating all personal identifiers. Multiple causes of death data were analyzed under a person-based approach, which eliminated duplication of counts of reported causes for each death [3]. Death records were classified based on tuberculosis reporting on any part of the death certificate corresponding to ICD10 codes A15-A19 (Tuberculosis block of three-character categories), B90 (Sequelae of tuberculosis three-character category) and B20.0 (HIV disease resulting in mycobacterial infection four-character subcategory) without mention of the former, as follows: tuberculosis not reported (TBNoR), tuberculosis reported as the underlying cause of death (TBUC) and tuberculosis reported as an associated cause of death (TBAC).

Age-standardized annual mortality rates for TBUC, TBAC and with TB reported (TBUC plus TBAC) were estimated for the State of Amazonas, using the direct standardization method and WHO 2000–2025 standard population [10].

Associated and underlying causes that related to TBUC and TBAC deaths, respectively, were listed in tabulation lists according to natural history of tuberculosis and frequency criteria.

Mortality odds ratios (OR) of reporting as TBUC and TBAC in relation to TBNoR were estimated using multinomial logistic regression modeling [11]. Covariates were added in order to adjust for potential confounding as follows: triennium in which death occurred (2006–2008, 2009–2011 and 2012–2014), place of residence prior to, and of occurrence of death (state capital–Manaus–and all other municipalities), gender (male and female), age group (0–14, 15–39, 40–64, 65 and over), race/skin color (white and nonwhite) and education level (0–7, 8–11, 12 and more school years and “not applicable”). The nonwhite race/skin color category was comprised of all categories available on the death certificates except white, therefore it included black, yellow, *mulatto* and indigenous. Education level was classified as not applicable for all children under six years of age in accordance with the Brazilian Education Policy. Missing data, interpreted as proxies of lack of data quality, was considered an additional category of all covariates (ignored) and kept in the models to investigate the association with TB reporting. In order to avoid inaccuracy due to the small sample, little missing data (less than 1,0%) were excluded.

The categories of the covariates were inserted as indicator (dummy) variables [12]. Statistical significance was set at the 20% and 5% levels, respectively, for the entry and retention of covariates in the model, and 95% confidence limits were calculated for final-model adjusted OR estimates. Statistical significance was established by the Wald test and the quality of fit of the final model by the analysis of deviance measures [11]. Analyses were developed using Stata 12 software (StataCorp. 2011. *Stata Statistical Software*: *Release 12*. College Station, TX: StataCorp LP).

In order to investigate potential bias related to single cause reporting due to ill-defined underlying causes, alternative models were fitted excluding any deaths due to ICD10 codes R98 (Unattended death) and R99 (Other ill-defined and unspecified causes of mortality) in the TBNoR group.

The study was approved by the Ethics in Research Board Committee (ERBC) of the *Fundação Alfredo da Mata Hospital*, Amazonas, under the National Ethics in Research Board Committee—CONEP (protocol number–CAAE—59417016.3.0000.0002; available at http://plataformabrasil.saude.gov.br/login.jsf). The ERBC waived the requirement for informed consent. All data were fully anonymized prior to the onset of the study.

## Results

Between the years 2006 and 2014, 120,701 death certificates were registered in the state of Amazonas. 331 records (0,27%) with missing data for gender (98; 0,08%) and/or age group (275; 0,23%—of whom 42 also lacked information about gender) were excluded, resulting in a study population of 120,370 death records. Tuberculosis was reported on 1,925 (1.6%) death certificates and was assigned as the underlying cause on 1,159 (60.2%) records, 1.5 times higher than when recorded as an associated cause (766; 39.8%).

Table 1 describes the study population according to categories of covariates and the study outcome. Assignment of tuberculosis as the underlying cause of death was higher when death occurred between 40 and 64 years of age and education level was unknown. Lower frequencies were observed under 15 years of age, of white race and increasing education level. Tuberculosis, when reported as an associated cause of death, increased within the state capital Manaus, either as place of residence or occurrence, in patients of ages between 15 and 39, 8 or more years of schooling and in cases where race/skin color and education level information was missing from the reports. Reporting was distinctively lower for the age groups of under age 15 and above 64.

**Table 1:** Distribution of death records according to categories of covariates and tuberculosis^§^ reporting as cause of death, state of Amazonas, Brazil, 2006 to 2014.

Covariates	Tuberculosis reporting on death certificate						Total	
	Underlying cause		Associated cause		Not reported			
	n	%	n	%	n	%	n	%
**Total**	**1,159**	**1.0**	**766**	**0.6**	**118,445**	**98.4**	**120,370**	**100.0**
Trienium								
2006–2008	342	1.0	200	0.6	35,088	98.5	35,630	100.0
2009–2011	406	1.0	267	0.7	39,316	98.3	39,989	100.0
2012–2014	411	0.9	299	0.7	44,041	98.4	44,751	100.0
Place of residence								
All other municipalities	420	0.9	115	0.3	43,749	98.8	44,284	100.0
State capital (Manaus)	739	1.0	651	0.9	74,696	98.2	76,086	100.0
Gender								
Male	747	1.0	511	0.7	71,298	98.3	72,556	100.0
Female	412	0.9	255	0.5	47,147	98.6	47,814	100.0
Age group (years)								
0–14	33	0.2	5	0.0	16,131	99.8	16,169	100.0
15–39	223	1.0	379	1.7	21,762	97.3	22,364	100.0
40–64	420	1.4	261	0.8	30,352	97.8	31,033	100.0
65 and over	483	1.0	121	0.2	50,200	98.8	50,804	100.0
Race/skin color								
White	158	0.7	131	0.6	21,944	98.7	22,233	100.0
Non-white	960	1.0	601	0.6	92,926	98.3	94,487	100.0
No answer	41	1.1	34	0.9	3,575	97.9	3,650	100.0
Education level (years of schooling)								
0–7	822	1.1	450	0.6	72,970	98.3	74,242	100.0
8–11	124	0.8	160	1.0	15,687	98.2	15,971	100.0
12 and more	29	0.6	45	0.9	5,023	98.5	5,097	100.0
Not applicable*	15	0.1	4	0.0	13,993	99.9	14,012	100.0
No answer	169	1.5	107	1.0	10,772	97.5	11,048	100.0
Place of ocurrence								
All other municipalities	325	0.9	55	0.1	37,469	99.0	37,849	100.0
State capital (Manaus)	834	1.0	711	0.9	80,976	98.1	82,521	100.0

^§^ Tuberculosis reporting on any section of the death certificate corresponding to ICD10 codes A15-A19 (Tuberculosis block of three-character categories), B90 (Sequelae of tuberculosis three-character category) and B20.0 (HIV resulting in mycobacterial infection four-character subcategory)—International Statistical Classification of Diseases and Related Health Problems 10th Revision [8].

*Education level was classified as “not applicable” for all children under six years of age according to Brazilian Education Policy.

**Fig 1:**
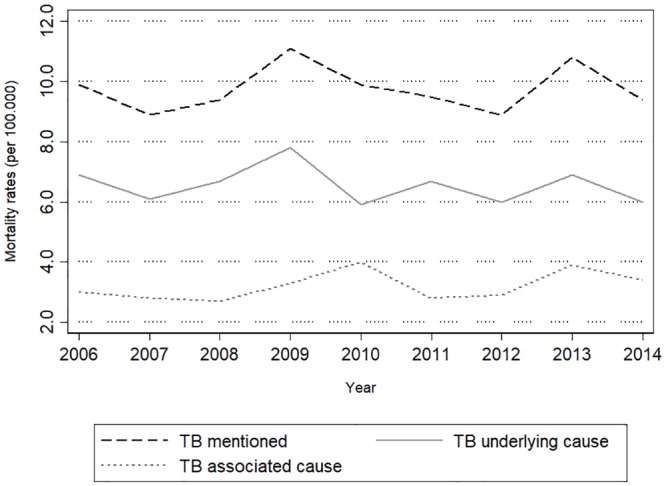
. Age adjusted* annual tuberculosis^§^ mortality rates from 2006 to 2014, State of Amazonas, Brazil. *Direct standardization method and WHO 2000–2025 standard population [10]. ^§^ Reporting of tuberculosis on any part of the death certificate, corresponding to ICD10 codes A15-A19 (Tuberculosis block of three-character categories), B90 (Sequelae of tuberculosis three-character category) and B20.0 (HIV resulting in mycobacterial infection four-character subcategory)—International Statistical Classification of Diseases and Related Health Problems 10th Revision [8]. TB reported: Tuberculosis reported as underlying or associated cause of death; TBUC: Tuberculosis reported as the underlying cause of death; TBAC: tuberculosis reported as an associated cause of death.

Age-standardized annual TBUC, TBAC, and TB reported mortality rates are presented in Fig 1. TBUC mortality ranged from 5.9 to 7.8/100,000, peaking in 2009 and remaining below 7.0/100,000 thereafter. TBAC mortality varied from 2.7 to 4.0/100,000 –approximately half of TBUC mortality range–peaking in 2010 (4.0/100,000) and 2013 (3.9/100,000). Overall mortality from TB when reported ranged between 8.9 and 11.1/100,000, which exceeded 10/100,000 only in 2009 (11.1/100,000) and 2013 (10.8/100,000).

All forms of active tuberculosis (A15-A19), the most frequent form of tuberculosis reporting (1.19%; n = 1,436), were 2.7 times more likely to be coded as the underlying cause of death. The term “Sequelae from tuberculosis” (B90) was reported on 189 (0.16%) death certificates and was 1.6 times more likely to appear as the underlying cause. HIV resulting in mycobacterial infection (B20.0), without mention of ICD10 codes A15-A19 or B90, was found in 308 death records (0.26%), and always as the associated cause.

Among deaths where tuberculosis was reported as the underlying cause of death (1,159), 941 (81.2%) cases were due to tuberculosis of the respiratory tract, 31 (2.7%) of the nervous system, 28 (2.4%) of all other organs, 45 (3.9%) to miliary tuberculosis and 114 (9.8%) to sequelae from tuberculosis.

Most of the associated causes mentioned, where tuberculosis was reported as the underlying cause (Table 2 –left side), may be interpreted as terminal, such as respiratory failure and sepsis–which were mentioned on more than 30% of death certificates–and renal failure. Other causes include types of pneumonia, nutritional and metabolic disorders–predominantly malnutrition and diabetes mellitus -, diseases of the respiratory, cardiovascular and digestive systems–chronic lower respiratory, lung interstitium, hypertensive and liver diseases—and mental disorders—psychoactive substance use, mainly alcohol and tobacco use.

**Table 2:** Distribution of associated causes on death certificates which reported tuberculosis as the underlying cause of death, and underlying causes on death certificates which reported tuberculosis as an associated cause of death, Amazonas State, Brazil, 2006 to 2014^§^.

Rank	Associated causes in death certificates reporting tuberculosis as underlying cause of death		%^†^	Underlying causes of death in death certificates reporting tuberculosis as associated cause		%^‡^
	ICD10* Chapter / Block of three-character categories / Three-character categories			ICD10* Chapter / Block of three-character categories / Three-character categories		
1	X	J96 Respiratory failure, not elsewhere classified	42.8	I	B20-B24 Human immunodeficiency virus [HIV] disease	68.4
2	X	J12-J18 Pneumonias	30.6	X	J40-J47 Chronic lower respiratory diseases	6.4
3	I	A40-A41 Sepsis	30.2	II	C30-C39 Malignant neoplasms of respiratory and intrathoracic organs	2.5
4	IV	E40-E46 Malnutrition	11.5	IV	E40-E46 Malnutrition	2.1
5	X	J40-J47 Chronic lower respiratory diseases	10.6	IX	I60-I69 Cerebrovascular diseases	1.7
6	XIV	N17-N19 Renal failure	7.0	XI	K70-K77 Diseases of liver	1.7
7	X	J80-J84 Other respiratory diseases principally affecting the interstitium	6.7	IX	I30-I52 Other forms of heart disease	1.6
8	IV	E10-E14 Diabetes mellitus	5.3	IX	I20-I25 Ischaemic heart diseases	1.3
9	IX	I10-I15 Hypertensive diseases	4.8	II	C15-C26 Malignant neoplasms of digestive organs	1.0
10	IX	I30-I52 Other forms of heart disease	4.2	IV	E10-E14 Diabetes mellitus	1.0
11	XI	K70-K77 Diseases of liver	3.6	IX	I10-I15 Hypertensive diseases	1.0
12	V	F10-F19 Mental and behavioural disorders due to psychoactive substance use	3.4	X	J80-J84 Other respiratory diseases principally affecting the interstitium	1.0

^§^ Tuberculosis reporting on any part of the death certificate corresponding to ICD10 codes A15-A19 (Tuberculosis block of three-character categories), B90 (Sequelae of tuberculosis three-character category) and B20.0 (HIV disease resulting in mycobacterial infection four-character subcategory).

* International Statistical Classification of Diseases and Related Health Problems, 10th Revision [8].

^†^ Proportion (%) of death certificates reporting each associated cause of death relative to all deaths due to tuberculosis (n = 1,159).

^‡^ Proportion (%) of death certificates with tuberculosis reported as associated cause of death (n = 766).

When tuberculosis was an associated cause, seven out of ten deaths were due to infectious diseases, almost exclusively to HIV (Table 2 –right side). Respiratory diseases, neoplasms, and cardiovascular diseases together accounted for approximately 16.5% of deaths, mainly due to chronic lower respiratory diseases (6.4%), cancer of respiratory and intrathoracic organs (2.5%) and hypertension-related diseases (cerebrovascular, ischemic and hypertensive diseases– 4.0%), respectively. Other relevant causes include malnutrition, liver diseases, and diabetes mellitus, together accounting for 5% of all deaths.

Seven of the top twelve causes listed in either side of Table 2 –namely malnutrition, diabetes mellitus, chronic lower and interstitium-related respiratory diseases, hypertensive and other forms of heart diseases, and liver diseases—were assigned as underlying or associated cause of death on death certificates which reported tuberculosis indistinctively.

Following univariate regression, all covariates met the statistical criteria (p<0,20 for at least one of the outcome categories) for entering multivariate modeling (S1 Table). Unadjusted OR for tuberculosis reporting as an associated cause was distinctively higher at ages 15 to 39 and 40 to 64, followed by 65 years and over–which was expressive, although lower OR estimates were found regarding TB as an underlying cause as well.

Adjusted OR for reporting TBUC (versus TBNoR) were significantly lower among the deaths of state capital residents (35% decrease), women (12% decrease) and with increasing education level (33% and 50% decrease, respectively, with 8 to 11 and 12 or more school years). When the education level criteria did not apply (among children aged 0 to 5 years) the chance of tuberculosis as the underlying cause decreased by 86%. Higher odds of TBUC were observed when deaths occurred among non-whites (36% increase) and in the state capital (71% increase) (see Table 3).

**Table 3:** Adjusted odds ratios for reporting tuberculosis^§^ as an underlying and associated cause of death, State of Amazonas, Brazil, 2006 to 2014.

Covariates	Tuberculosis reported as:					
	Underlying cause			Associated cause		
Trienium	OR	95% CI†		OR	95% CI†	
2006–2008	1.00	---	---	1.00	---	---
2009–2011	1.07	0.92	1.23	1.21	1.00	1.46
2012–2014	0.96	0.83	1.11	1.22	1.01	1.46
Place of residence						
All other municipalities	1.00	---	---	1.00	---	---
State capital (Manaus)	0.65	0.53	0.80	0.89	0.69	1.16
Gender						
Male	1.00	---	---	1.00	---	---
Female	0.88	0.78	0.99	1.01	0.86	1.18
Age group (years)						
0–14	1.00	---	---	1.00	---	---
15–39	1.39	0.86	2.26	35.89	5.06	255.94
40–64	1.87	1.16	3.01	16.55	2.32	118.11
65 and over	1.28	0.80	2.06	5.01	0.70	35.90
Race/skin color						
White	1.00	---	---	1.00	---	---
Non-white	1.36	1.15	1.62	0.94	0.77	1.15
No answer	1.50	1.06	2.13	1.30	0.88	1.91
Education level (years of schooling)						
0–7	1.00	---	---	1.00	---	---
8–11	0.67	0.55	0.81	0.97	0.80	1.16
12 and more	0.50	0.34	0.72	0.91	0.67	1.24
Not applicable*	0.14	0.07	0.27	0.71	0.08	6.36
No answer	1.38	1.16	1.64	1.66	1.33	2.07
Place of ocurrence						
All other municipalities	1.00	---	---	1.00	---	---
State capital (Manaus)	1.71	1.37	2.14	5.63	3.93	8.08
Constant	0.01	0.00	0.01	0.00	0.00	0.00

^§^ Tuberculosis reporting on any part of the death certificate, corresponding to ICD10 codes A15-A19 (Tuberculosis block of three-character categories), B90 (Sequelae of tuberculosis three-character category) and B20.0 (HIV resulting in mycobacterial infection four-character subcategory)—International Statistical Classification of Diseases and Related Health Problems - 10^th^ Revision [8].

^†^ Confidence interval.

* Education level was classified as “not applicable” for all children under six years of age according to Brazilian Education Policy.

The adjusted OR for reporting tuberculosis as associated cause increased in the last two trienniums (21% and 22%, respectively, in years 2009 to 2011 and 2012 to 2104), with adult ages (35.9 and 16.5 times higher, respectively, within ages 15 to 39 and 40 to 64) and when death occurred in the state capital (5.6 times higher) (Table 3).

No significant changes were noted following alternative models excluding deaths due to single cause ICD10 three-character categories R98 (Unattended death– 10,193 records) and R99 (Other ill-defined and unspecified causes of mortality– 6,628 records) in the TBNoR group (S2 Table).

## Discussion

Although predominantly assigned as the underlying cause, tuberculosis reporting as associate cause corresponded to 40% of all mentions of tuberculosis mortality in the State of Amazonas from 2006 to 2014. Deaths which mentioned tuberculosis as an associated cause are not usually counted in primary statistics of mortality. Considering all death certificates on which tuberculosis was mentioned, an average 50% increase in annual tuberculosis-related mortality rates was observed when compared with TBUC mortality rates. Similar patterns of tuberculosis reporting on death certificates were observed elsewhere in Brazil [4,13–15].

Causes of death associated with tuberculosis were mainly respiratory, infectious, nutritional, metabolic, cardiovascular and digestive diseases–frequently terminal diseases. Such causes are among those characterized as complications of tuberculosis. Improvements in the diagnosis and treatment of tuberculosis patients in a timely manner could be reached by enhancing primary health care, better training of health personnel, and screening for symptomatic respiratory cases, which are among the main measures necessary to avoid the occurrence of these worse outcomes [16].

The high frequency of tuberculosis-related mortality which is associated with malnutrition, diabetes mellitus, alcoholism and smoking found in the present study are in accordance with other reports in the related scientific literature. These findings reflect the role of emerging risk factors such as chronic diseases, socio-economic and behavioral aspects in increasing unsuccessful TB treatment outcomes [17]. Knowledge of these causes is important in order to control and prevent tuberculosis-related deaths, through timely diagnostic of the comorbidities, as well as for creating public health prevention and promotion policies (smoking cessation treatments, food safety, lowering alcohol consumption, etc.).

Mortality due to tuberculosis is associated with poor socioeconomic conditions (living outside Manaus, non-white race/skin color and low education level) and negatively related to the female sex. The higher odds of dying due to tuberculosis among non-whites, including natives of indigenous origin, and TB’s inverse relationship with education level, may reflect health-related social inequalities. These findings corroborate the data from literature, which shows the relationship between TB and socioeconomic deprivation and ethnic status in the state of Amazonas [18]. Similarly, the lower odds of tuberculosis being reported as the underlying cause of death, observed among deaths of residents in the State capital, may be explained by these patients having a higher socioeconomic level and better health care access when compared to the remaining municipalities. The association of male gender and poor socioeconomic conditions with high tuberculosis mortality has also been observed in other populations, reflecting the effects of both behavioral (non-adherence to treatment, alcoholism, smoking, etc.) and demographic (low access to health system and food safety, unemployment, precarious housing, etc.) characteristics on the outcome [19–21]. Public health and social policies as income distribution are measures that could impact these factors and TB mortality.

Mortality through tuberculosis was positively associated with adult ages, reflecting its strong relationship as an associated cause with HIV [15]. HIV infection was the most frequent underlying cause of death when tuberculosis was reported as an associated cause, followed by diseases of the respiratory and cardiovascular systems, neoplasms, nutritional and metabolic disorders, and digestive diseases. Similar cause-specific mortality profiles were found in two cohort studies involving tuberculosis patients in Brazil and China [14,22].

The high frequency of deaths due to AIDS with mention of tuberculosis may reflect the natural history of HIV disease in high tuberculosis prevalence settings, as in the State of Amazonas. However, it may also suggest the inadequate implementation of the Brazilian Ministry of Health’s guidelines for HIV disease control, such as early HIV infection diagnosis, latent tuberculosis infection screening and preventive therapy among HIV patients. In Brazil, HIV is commonly diagnosed following the onset of tuberculosis and the association of lack of isoniazid preventive therapy [23].

The increasing odds of tuberculosis being reported as an associated cause suggest that in recent years an overall improvement in diagnosis has occurred, more specifically of HIV and related comorbidities. In a recent study, the linking of tuberculosis and HIV/Aids case report databases reduced underreporting and improved the epidemiological surveillance of the two diseases in the state of Amazonas [24]. Also,assignment of cause of death has improved in Amazonas state, with decreasing trends in reporting mortality with ill-defined and unspecified causes [25].

The higher odds of reporting tuberculosis when death occurred in the State capital, which showed to be even higher as an associated cause, may reflect the concentration of health care facilities in Manaus, including reference health services for diagnosis and treatment of infectious and non-infectious diseases. This hypothesis is supported by studies that have shown that the state capital presents better levels of performance of the health services [24,26].

Tuberculosis remains a significant health burden in developing countries. Notwithstanding all efforts aimed at its control and the increasing coverage of primary health care units in Brazil from 2006 to 2014, high incidence rates are expected for future decades as a consequence of high prevalence of *Mycobacterium tuberculosis* infection following present and past high transmission rates. However, deaths due to tuberculosis are, in most cases, avoidable once access to prompt and adequate health care is available [14].

Among the limitations of our study, cause of death records, the only data source used, may lack sensitivity and specificity required for medical outcomes, more specifically for tuberculosis diagnosis [14,27]. Although SIM has systematically improved during the past decades, it still lacks completeness, better cause of death information and additional variables for death assessment, especially in the Northern regions of Brazil—including the State of Amazonas–where proportional mortality due to ill-defined causes, although presenting a declining trend, remains high [28]. Despite all technical criteria regarding data analysis and variable selection, these limitations could influence the associations on both overestimation and underestimation.

Despite such limitations, our findings are consistent with the findings of other authors’ studies which were developed in different settings, time periods and other distinct populations. Also, predictors of tuberculosis reporting on death certificates remained unchanged, even after excluding deaths due to ICD10 codes for ill-defined causes.

## Conclusions

Tuberculosis mortality was reported as the most predominant underlying cause of death in the State of Amazonas, from 2006 to 2014. However, a substantial increase (50%) in mortality rate estimates followed the inclusion of death certificates in which tuberculosis was assigned as the associated cause.

Tuberculosis was concurrently reported on death certificates with a myriad of infectious and non-infectious diseases, as well as with ill-defined causes. Causes associated with tuberculosis when assigned as underlying cause of death were mainly related to clinical complications and exposure to risk factors. HIV was the main underlying cause of deaths in which tuberculosis appeared as the associated cause.

Tuberculosis, when reported as the underlying cause of death, was associated with male gender and indicators of unfavorable socioeconomic conditions, as well as health care access constraints, whereas when reported as an associate cause, it was related to adult age groups, typically related to high HIV disease incidence, and occurrence in recent years, due to improvements in health care and better assignment of cause of death. The occurrence of death from TB in Manaus, the State capital, positively predicted both types of tuberculosis mortality reporting–more strongly as an associated cause which is possibly due to higher coverage and better performance of health care and diagnostic facilities.

## Supporting information

S1 Table Unadjusted odds ratios of reporting tuberculosis^§^ as underlying and associated cause of death, State of Amazonas, Brazil, 2006 to 2014. (DOCX)

S2 Table Adjusted odds ratios of reporting of tuberculosis^§^ as underlying and associated cause of death after excluding deaths due to ill-defined single causes^¥^, State of Amazonas, Brazil, 2006 to 2014. (DOCX)
