# Correction: Social, demographic, health care and co-morbidity predictors of tuberculosis mortality in Amazonas, Brazil: a multiple cause of death approach

**DOI:** 10.1371/journal.pone.0229749

**Published:** 2020-02-21

**Authors:** Vanderson de Souza Sampaio, Maria Gabriela de Almeida Rodrigues, Leila Cristina Ferreira da Silva, Daniel Barros de Castro, Patrícia Carvalho da Silva Balieiro, Ana Alzira Cabrinha, Antonio José Leal Costa

There are errors in the Funding statement. The correct Funding statement is as follows: This work was supported by grant Programa Estratégico de Ciência, Tecnologia & Inovação nas Fundações de Saúde–PECTI/AM/SAÚDE, Carta Convite 001/2014 and PAPAC, EDITAL N° 005/2019, both of Fundação de Amparo à Pesquisa do Estado do Amazonas—FAPEAM. The funders had no role in study design, data collection and analysis, decision to publish, or preparation of the manuscript.
