# Supplementary material for: Social, demographic, health care and co-morbidity predictors of tuberculosis mortality in Amazonas, Brazil: a multiple cause of death approach
Source: PLoS One. 2020 Jan 29;15(1):e0218359. doi: 10.1371/journal.pone.0218359 (PMC6988942; doi:10.1371/journal.pone.0218359)
Supplement: S1 Table — (DOCX) [file pone.0218359.s001.docx]

**Table S1 - Unadjusted odds ratios of reporting tuberculosis^§^ as underlying and associated cause of death, State of Amazonas, Brazil, 2006 to 2014**

^§^ Tuberculosis reporting in any part of the death certificate corresponding to ICD10 codes A15-A19 (Tuberculosis block of three-character categories), B90 (Sequelae of tuberculosis three-character category) and B20.0 (HIV resulting in mycobacterial infection four-character subcategory) - International Statistical Classification of Diseases and Related Health Problems - 10^th^ Revision [8].

† Confidence interval.

± p ≤ 0,20 – calculated using the Wald test.

*Education level was classified as “not applicable” for all children under six years of age according to Brazilian Education Policy.
